# Contract Research Organizations (CROs) in China: integrating Chinese research and development capabilities for global drug innovation

**DOI:** 10.1186/s12992-014-0078-4

**Published:** 2014-11-19

**Authors:** Yun-Zhen Shi, Hao Hu, Chunming Wang

**Affiliations:** State Key Laboratory of Quality Research in Chinese Medicine, Institute of Chinese Medical Sciences, University of Macau, Macao, China

**Keywords:** Contract research organization, CRO, Pharmaceutical R&D, Global integration, China

## Abstract

The significance of R&D capabilities of China has become increasingly important as an emerging force in the context of globalization of pharmaceutical research and development (R&D). While China has prospered in its R&D capability in the past decade, how to integrate the rising pharmaceutical R&D capability of China into the global development chain for innovative drugs remains challenging. For many multinational corporations and research organizations overseas, their attempt to integrate China’s pharmaceutical R&D capabilities into their own is always hindered by policy constraints and reluctance of local universities and pharmaceutical firms. In light of the situation, contract research organizations (CROs) in China have made great innovation in value proposition, value chain and value networking to be at a unique position to facilitate global and local R&D integration. Chinese CROs are now being considered as the essentially important and highly versatile integrator of local R&D capability for global drug discovery and innovation.

## Background

China has prospered in its pharmaceutical research and development (R&D) capability in the past decade [1-4]. The Chinese government has invested massively to support drug development in the country since the establishment of “New Drug Creation and Development programme” in 2008 [5]. In 2012, the State Council issued “12th Five-Year Development Plan for National Strategic Emerging Industries” which strategically positioned biomedicine as a key emerging industry [6]. The overwhelming support has not only strengthened the research teams and upgraded laboratory facilities in the local institutes, but also attracted many Chinese scholars overseas to return and establish their own technology firms in China. These efforts have greatly contributed to the emerging pharmaceutical R&D capabilities in China [7-9].

Despite the favorable conditions, challenges remain in how to integrate the rising pharmaceutical R&D capability of China into the global development chain for innovation of new drugs [5]. Multi-national corporations (MNCs) and research organizations overseas often find it difficult to integrate China’s R&D capabilities into their own. For instance, there are excessive government restrictions on foreign organizations which intend to get or are already involved in government-funded projects. On the other hand, local academic researchers rely heavily on government funding to sustain their work and focus primarily on publishing academic papers to build up their academic reputation. Cooperation with organizations overseas on industrial drug discovery is not usually their top priority. Moreover, local pharmaceutical companies can be highly skeptical about the motivation of foreign pharamceutical companies seeking R&D cooperation, which prevents mutual consensus on establishing collaboration and always results in failures of joint venture and cooperation projects.

In China, the contract research organization (CRO) sector booms as they are expected to become a feasible channel to facilitate the integration of local R&D capabilities into global discovery and development [10-13]. Since 2008, we have started to investigate the Chinese CROs by collecting data from multiple sources including conducting field interviews with CRO managers and research staffs, attending industrial conference of CRO, and collecting archival documents of CROs from the company websites and public bibliographic databases. Until the end of 2013, we collected substantial data about 66 leading Chinese CROs. In addition, we also interviewed 12 foreign pharmaceutical company managers, 10 local university researchers and 8 policy makers. All the data collected from the various sources indicated that Chinese CROs were being considered as an essentially important and highly versatile integrator of local R&D capability for global drug discovery and innovation.

### Evolution of Chinese CROs

The CRO industry in China began when *Quintiles* set up its Hong Kong Office and *MDS Pharma Service* started an investment company in Beijing in 1996. A key opportunity for the CROs to prosper appeared in 1998 when the Chinese government implemented new drug registration regulations which mandated pharmaceutical companies to submit scientific evidence to prove safety and efficacy of new drugs. In response to these regulatory requirements, both local and global pharmaceutical companies were in immediate need of agents that could provide R&D services. Currently, there are about 300 CROs in China which provide preclinical and clinical research services for MNCs, local companies, as well as other R&D organizations such as universities and academic institutes.

While China-based CROs only started in the clinical trial arena, they entered the market of preclinical research very soon with their significance increasing rapidly [10]. By 2012, there were more CROs focusing on preclinical research service than those exclusively on clinical trials. After the global financial crisis in 2008, there was even a trend of outsourcing clinical trial services provided in China. Under the influences of dynamic market fluctuation and uncertainties in regulatory framework, industrial consolidation of Chinese CROs happened dramatically through mergers and acquisitions (M&A). Consequently, many small Chinese CROs vanished, leaving a handful of local leading CROs to become the key players such as*WuXi AppTec*, *Tigermed*, and *VenturePharm*. In order to compete against the global giant CROs such as *Quintiles*, *Covance*, *PPD*, *ICON*, and *Parexel*, Chinese CROs had to create their unique business models. Our study clearly shows that in order to establish their standpoint, China’s CROs had innovated their business model in three aspects– value proposition, value chain and value networking. By doing so, they had successfully evolved to facilitate the integration of the nation’s R&D capabilities in response to the global challenge.

### From cost-saving to value-added proposition

Another distinctive observation revealed by our data is that, unlike the traditional image of cost-saving service imposed by the notion of “made-in-China”, Chinese CROs had changed their value proposition from just cost-saving to value-added for clients. When the Chinese CROs began their business at the beginning of 21^st^ century, most of them strived to survive by attracting clients with their advantages of low human and material cost without too much emphasis on the service quality. However, the ruthless “red ocean” competition on price and tough demands from high-end clients eventually forced CROs with weak R&D and management capability out of the market rapidly. The surviving CROs in China then sought new value propositions through three main aspects: i) they invested into technology improvement and applied the most advanced biotechnology into their R&D services; ii) they invested into R&D capacity building, installed the most advanced laboratory instruments and advanced laboratory facilities to ensure scale economy and high efficiency; iii) they adopted global R&D standards and guidelines into their practices to meet the international requirements including the Good Laboratory Practice (GLP), Good Manufacturing Practice (GMP), and Good Clinical Practice (GCP). All these efforts enabled Chinese CROs to employ a new, systemic value proposition which was recognized as an increasingly important quality sought after by their clients.

There were many examples to demonstrate the important transition of Chinese CROs to from “cost-saving” mindset to value-added proposition. The first one was *XBL-China*, a CRO located in Nanjing City and founded by returnee scholars from the United States [14]. This CRO had innovative capabilities in drug metabolism and bioanalytical method development and validation. In particular, it was equipped with world-class drug metabolism and pharmacokinetic (DMPK) research capabilities, and held a unique technology of using isotopes to trace the pharmacokinetic features of candidate drugs in clinical study. It also complied with the tGLP standards adopted by both U.S. Food and Drug Administration (FDA) and China Food and Drug Administration (CFDA) in order to ensure the technological specification of the company sufficiently meet the international quality standards, and thereby guarantee efficiency and competency. Today, this China-based CRO is among all the first choice for both local and international clients which require isotopic tracing technique.

Another successful example was *Pharmaron* which was a preclinical CRO founded in 2003 by Chinese returnees who were highly experienced researchers in pharmaceutical MNCs. In the beginning, this CRO operated in accordance with the mission statement “to provide the highest quality R&D services while helping our customers advance their projects in a timely and cost-effective manner” [15]. In addition to the strong pharmacochemistry capability, *Pharmaron* rapidly increased investment to acquire the most advanced biotechnology in order to enlarge its business scope from single business of chemical service for drug R&D to a series of preclinical services across a number of disciplines including chemistry, biology, DMPK, pharmacology, toxicology and chemical development. At present, *Pharmaron* is capable of conducting the leading preclinical research that meets global standards. Moreover, *Pharmaron* has developed an advanced information system that provides clients with accurate and timely traceability of their projects. Its core competence in R&D capability and information management has secured 7 of the top 10 pharmaceutical MNCs as its long-term clients.

Considering that the Chinese policy was determined to fully support the sustainable development of traditional Chinese medicine (TCM), some CROs such as *Bionovo* chose to focus primarily on TCM-related contract service [16]. Based on its accumulated capabilities in clinical trial study of TCM, this CRO aimed its market share on Phase I-IV clinical trial research for TCM. For this, *Bionovo* created a series of clinical research practices and guidelines to guide the practice of and ensure the quality of clinical research of TCM. Moreover, it provided additional value for its clients through promoting pharmaceutical economics of TCM which was also strongly encouraged by CFDA and State Administration of Traditional Chinese Medicine (SATCM) in recent years. Currently, it is able to provide over 300 clinical research services across 20 therapeutic areas, and is now expanding its business scope to consulting services. This company has become a leading TCM CRO taking up 60%-70% TCM clinical research market share in China.

As demonstrated in the above examples, with the new value propositions, the client scale and composition of Chinese CROs have undergone huge transformation. More and more local and global clients value and choose the contract services provided by Chinese CROs and no longer rely solely on the local academic organizations. Both global and local clients are keen to offer more technology-intensive and knowledge-oriented contracts to Chinese CROs, which in turn help to boost the development of R&D capabilities and profitability of Chinese CROs accordingly.

### Constructing a complete service chain

According to the data in our study, Chinese CROs were able to carry out contract service that covers the entire drug development value chain, ranging from early drug discovery to post-market reassessment. The services provided by Chinese CROs encompassed almost the entire outsourcing market. At the same time, each of these CROs had its own unique specialties and was determined to reinvent its internal key business processes to out-stand both local and overseas CROs.

There are many preclinical CROs in China which are of small scale facing throat-cut competition. In order to survive, they need to have the appropriate R&D capability to offer highly specialized services in the service chain that no other CROs can match. One of the examples was *Tigermed* which specialized in clinical trials, data management, biostatistics, regulatory affairs and medical translation [17]. For cost reduction reasons, some global CROs also relocated their clinical research business and established multi-center clinical trial laboratory in China. In this context, companies such as *Tigermed* set up an in-house department for regulatory affairs to offer customized regulatory services on pharmaceuticals, biological products, and medical devices. *Tigermed* was committed to accelerating medical product development with cost efficiency and quality. While clinical trial planning, management and implementation brought in the biggest profit for *Tigermed* accounting for 58.6% of the total sales in 2012, high gross margins were gained through both data management (66.61%) and regulatory affairs (61.39%). *Tigermed* was ready to expand and take a share of the global market. Recently, *Tigermed* participated in an international multi-center clinical research project run by Lilly on an anti-diabetic drug.

As the overall Chinese CROs market thrives, some large CROs take a step further to acquire an extended value chain. *WuXi AppTec*, one of the largest CROs in China, which used to operate only within the preclinical experiment business has begun to provide “one-stop service” with the full coverage from drug discovery to clinical & regulatory [18]. To do this, *WuXi AppTec* acquired *AppTec* (a famous American medical device company) in 2008 and established integrated service. On the contrary, *VenturePharm,* which started as a clinical CRO, had successfully developed to provide preclinical research services for big pharmaceutical companies.

### Bridging network

As shown by the data in our study, Chinese CROs enjoyed a great advantage of a unique integrated network of local universities/academic institutes, hospitals and domestic pharmaceutical firms. In any mature pharmaceutical sector around the world, pharmaceutical companies (especially MNCs) were often the major platform for drug R&D. In China, however, universities and research institutes were the primary R&D forces and the local pharmaceutical companies were still far from having sufficient innovative capabilities. They received most of the voluminous government financial investment and were never short of expert resources. The sophisticated connection of CROs with these university or research institutes was one great advantage over foreign and local pharmaceutical firms. Most CROs were in fact spin-offs from universities and academic institutes or were founded by Chinese returnees who studied in the corresponding university and still maintained good relationships with the academic staff. Therefore, they could have access easily to the well-equipped university laboratory and other advanced facilities. This was also why Chinese CROs were able to participate in many academic projects initiated by universities or researchers. In the same way, Chinese CROs were also able to invite prestigious and influential local researchers to provide professional help to uplift their internal R&D competencies. For instance, *RunDo* employed the executive president of China nonprescription medicines association and an academician of the Chinese Academy of Engineering as its academic advisors [19].

In 2012, there were 1,431 hospitals in China, of which 420 had GCP certifications and a rich source of patients enough for multiple clinical R&D studies. However, foreign pharmaceutical firms always had huge difficulties to get access to these hospitals and to initiate clinical research simultaneously at different locations [20]. On the contrary, Chinese CROs always had good relationships with the local hospitals. For example, *Tigermed* developed a cooperation network that covered most high-end hospitals to enable clinical research across China at the same time. Through a similar network with hospitals, many other Chinese CROs were also able to get involved in innovative medical and drug research projects. *Giant*, a clinical CRO in Beijing, was invited to participate a huge mass data project to document and analyze medical records from the most leading hospitals in China, which was initiated by the Chinese Academy of Medical Sciences [21].

Our study also showed that more and more local pharmaceutical firms also preferred the service provided by CROs in China. With the industrial policy and funding support from government, some local pharmaceutical firms also tried to develop their own R&D capabilities. During the process, they might need to turn to CROs for their professional help and even invite CROs to join their innovative R&D project run or supported by government. For example, *Medicilon*, a CRO founded by Chinese returnees, participated in several innovative drug projects run by local pharmaceutical companies and funded by the Chinese government.

CROs in China were also found to be influential to the implementation of price control on drugs initiated by the Chinese government. The health system in China was highly sophisticated and was exposed to significant pricing and market access (P&MA) [22]. In the last decade, the Chinese government continued to reinforce price control on medicine at the national and provincial level [23]. However, one of the major resistances in fact originated at hospital level. In order to facilitate the listing of their drugs products on the hospital formulary maintained by the in-house pharmacy, drug companies needed to somehow get involved in the hospital listing processes. To act as a bridge, CROs could also play a role favorable to the drug companies and provide them with important insights of government policy and the implicit rules of pricing and market access of the corresponding hospital.

### Integrator position of Chinese CROs

As discussed above, Chinese CROs had been trying to evolve their business model to outshine themselves at the international level. They successfully established a unique position as a facilitator to integrate Chinese local R&D capabilities through their collective and individual efforts in three major aspects: (1) development of the in-house R&D capabilities; (2) specialization and extension of their technical roles throughout the entire value chain; and (3) establishment of a competency network involving local research units (universities, academic institutes, and hospitals) and pharmaceutical firms. By employing their growing R&D capabilities and their unique network with the major players in the drug sector in China, CROs were able to provide feasible and efficient means for global players to enter China’s market and, at the same time, integrate Chinese pharmaceutical R&D capabilities into the global drug industry.

In order to optimize productivity and efficiency of a research project, global pharmaceutical companies might need to carefully design their strategies when acquiring local R&D capabilities [24]. Taking into account the rising pharmaceutical R&D capabilities in China, seeking help from the Chinese CROs, which was often associated with more benefits and less risks, was preferred by the global companies over joint venture or cooperation of any form. As shown in Table 1, participation of CRO in the business could generate more benefits in terms of technology acquisition, experimental efficiency and cost saving as CRO was usually less constrained by complex operation procedures and finance and technology exchange negotiation. While joint venture could offer high level of technology acquisition and be relatively cost saving, there were always antagonizing forces of complicated administration coordination between joint venture partners which could result in low experiment efficiency. Moreover, due to loose cooperation agreement and heavy negotiation burden, the benefits of technology acquisition, experimental efficiency and cost saving through cooperation agreement with local R&D organizations were usually limited for foreign organizations.

**Table 1:** **Benefits and threats for foreign organization: CRO, joint venture and cooperation**

	**CRO**	**Joint venture**	**Cooperation**
**Benefits**			
Technology acquisition	High	High	Low
Experimental efficiency	High	Low	Low
Cost saving	High	Middle	Low
**Threats**			
Intellectual property risk	Low	High	High
Coordination difficulty	Low	High	High
Policy restriction	Low	High	Middle

In addition, joint venture and cooperation agreement could also be hindered by concerns over possible intellectual property leak, government regulation, and coordination cost. CROs could provide legal and business-based mechanisms to protect foreign pharmaceutical companies from these institutional threats [25]. This additional function of CROs was strongly encouraged and supported by the Chinese government as part of the CRO industrial development, unlike joint venture and cooperation agreement which might have to undergo stringent government review for approval in many cases. The risks of intellectual property leak and possible coordination conflicts were comparatively lower as the business contracts between CROs and foreign organizations were usually clear and specific.

### Future perspectives

In summary, our study demonstrated that Chinese CROs had evolved to be the most active component in the Chinese pharmaceutical R&D arena. They had created not only increasing business opportunities for themselves but also generated extensive industrial impact for both global and local pharmaceutical R&D.

As a service-oriented industry, CROs in China had been dedicated to accommodate and fulfill their clients’ demands [26]. The Chinese CROs were considered as the most efficient channel for foreign organizations to obtain and use the emerging pharmaceutical innovation capabilities in China. In particular for some MNCs, they now realized the unique benefits of the services provided by Chinese CROs and were keen on establishing closer relationships with them. Some companies even set up Asian research teams in China to implement and coordinate their research strategies with Chinese CROs.

Simultaneously, Chinese CROs made great contributions to the development of national pharmaceutical R&D through their interactions with local academic units and pharmaceutical companies. Our study also showed that while Chinese CROs endeavored to improve their R&D capabilities, they also acquired R&D management practices through their business relationship with global clients. This knowledge transfer did not stop within the CROs but extended to the local universities and pharmaceutical companies through their sophisticated linkage with CROs. As a result, Chinese CROs reshaped the R&D configuration of the national pharmaceutical sector and exerted great impact on the overall pharmaceutical sector in China.

Chinese CROs were also found to be very prudent in terms of avoiding conflicts between cost and service. Some of them even changed the practice standards of employing local resources in order to minimize the risks. For example, *Pharmaron* no longer relied merely on returnees from overseas and started to recruit local researchers with Ph.D. degree who asked for less compensation. It also established alliances with several famous laboratories of the local universities to get access to their advanced laboratory instruments and facilities with reasonably low cost. In addition, *Pharmaron* was able to apply for government funding that was specifically set for high-tech companies like CROs. By all these means, *Pharmaron* and other CROs alike in China were able to carefully maintain the service quality at a lower cost.

It was considered highly important by the CROs in China to meet the high expectation on quality control for both local and international clients. While purchasing expensive laboratory instruments was relatively easy, how to improve the internal management and operation process to increase quality control remained a major challenge for the Chinese CROs. In view of shortage of appropriate local knowledge input, Chinese CROs had to learn from the international CRO management and practice guidelines adopted overseas. For example, *XBL-China* was certified by the Association for Assessment and Accreditation of Laboratory Animal Care (AAALAC) to indicate its capability in quality control for animal experiments. In addition, some CROs established separate data management departments to ensure the accuracy and integrity of experimental data.

Between 2001 and 2013, there were 19 policies and regulations specific to CROs implemented by the Chinese government. These policies and regulations directly contributed to the development and industry integration of China-based CROs. For example, many pharmaceutical MNCs did not want to outsource their Phase I to China and Chinese CROs could just conduct Phase II-III clinical trials due to long administration process of government and hospitals. The government, therefore, dispatched the “Guiding Principle for Administration on Phase I Clinical Trials on Drugs” in 2011 to better regulate and streamline first-in-human clinical trials. Moreover, the rising venture capital in China was paying more attention to the CROs with advanced biotechnologies [27]. With a continuous emphasis on drug innovation and global demand for innovative capabilities, Chinese CROs are prepared to take up increasingly important role as nexus of both external and internal pharmaceutical R&D efforts, and play a critical role in the pharmaceutical industry in China.
